# Preoperative psychological competitive ability has little relationship with subjective knee function and return to sports at 6 months postoperatively in patients with anterior cruciate ligament reconstruction

**DOI:** 10.1016/j.asmart.2023.10.001

**Published:** 2023-11-06

**Authors:** Takuya Sengoku, Junsuke Nakase, Rikuto Yoshimizu, Mitsuhiro Kimura, Tomoyuki Kanayama, Goro Sakurai, Shinya Yoshida, Takashi Kitagawa, Katsuhiko Kitaoka, Hiroyuki Tsuchiya

**Affiliations:** a Section of Rehabilitation, Kanazawa University Hospital, 13-1 Takaramachi, Kanazawa, 920-8641, Japan; b Department of Orthopaedic Surgery, Graduate School of Medical Sciences, Kanazawa University, 13-1 Takaramachi, Kanazawa, 920-8641, Japan; c Department of Physical Therapy, School of Health Science, Shinshu University, 3-1-1 Asahi, Matsumoto, Nagano, 390-8621, Japan; d Department of Orthopaedic Surgery, Kijima Hospital, Matsuteramachi, Kanazawa, Ishikawa, 920-0011, Japan

**Keywords:** Anterior cruciate ligament, Knee injuries, Psychological factors, Return to practice, Subjective knee function

## Abstract

**Objective:**

This prospective study aimed to investigate the relationship between preoperative psychological competitive ability and preoperative and 6 months postoperative subjective knee function in patients undergoing anterior cruciate ligament (ACL) reconstruction.

**Methods:**

Eighty-four patients who underwent ACL reconstruction and had a Tegner Activity Scale score of 6 or more were included in this study. Preoperatively, all patients were administered the Diagnostic Inventory of Psychological Competitive Ability for Athletes (DIPCA.3) for psychological competitive ability assessment and the International Knee Documentation Committee (IKDC) Subjective Knee Evaluation Form for subjective knee function assessment. The IKDC subjective score was re-evaluated 6 months postoperatively. We evaluated the associations of volition for competition, mental stability and concentration, confidence, strategic ability, and cooperation (DIPCA.3 factors) with the IKDC subjective score preoperatively and 6 months postoperatively. Furthermore, patients were classified into two groups according to whether they could return to participate in the entire practice and compared the DIPCA.3 total score and IKDC subjective score.

**Results:**

The DIPCA.3 confidence score negatively correlated with the preoperative IKDC subjective score (β = −0.34, p = 0.04). However, there was no association between the DIPCA.3 score for all variables and IKDC subjective score at 6 months after ACL reconstruction. Moreover, the DIPCA.3 total score was not related to return to participate in the entire practice 6 months after ACL reconstruction.

**Conclusion:**

Psychological competitive ability is not associated with a return to participate in the entire practice, and a low preoperative IKDC subjective score should not be viewed too negatively.

## 1 Introduction

The psychological state is one of the most important factors related to return to sport after anterior cruciate ligament (ACL) reconstruction.^1^, ^2^, ^3^, ^4^, ^5^, ^6^ Ardern et al.^7^ reported that the return-to-sport rate after ACL reconstruction is very low, with 65 % of patients achieving their preoperative activity level and 55 % achieving a competitive level. Improving this rate is a major challenge, but a detailed psychological state assessment may provide a boost.

The Diagnostic Inventory of Psychological Competitive Ability for Athletes (DIPCA.3) is an assessment tool developed in Japan to diagnose psychological competitive ability as a general characteristic required by athletes in competitive situations.^8^^,^^9^ Psychological competitive ability as used in this assessment tool, is commonly defined as mental strength, and the ability to demonstrate one's skills in a competitive match.^10^^,^^11^ It is available in many languages and can be used worldwide.^12^^,^^13^ The psychological factors included in the DIPCA.3, volition for competition, mental stability and concentration, confidence, strategic ability, and cooperation are graded on a 5-point scale. The higher the score, the better the athlete's psychological competitive ability. Based on the results of the DIPCA.3, psychological training can create an excellent psychological state in a competition and create a mentality that allows you to demonstrate your abilities.^9^^,^^14^ Most reports on psychological status to date have focused on the postoperative state.^3^^,^^4^^,^^6^ In addition, competitive ability is usually classified according to activity level and sport. By using DIPCA.3, however, it is possible to classify athletic performance according to the psychological aspects of the individual, independent of the sport. In a study evaluating ACL injury using the DIPCA.3, higher scores on volition for competition and strategic ability have been reported to be related with ACL injury.^15^

The International Knee Documentation Committee (IKDC) Subjective Knee Evaluation Form is commonly used to assess subjective knee function after an ACL injury.^16^ It is used to investigate knee-related symptoms and knee function,^17^ and many reports have shown its usefulness for evaluating a return to sport after ACL reconstruction.^18^, ^19^, ^20^ IKDC subjective score has also been reported to correlate with the ACL-Return to Sports after Injury score (ACL-RSI), which has been used frequently in recent years.^21^ Although the ACL-RSI is an assessment tool to check psychological readiness to return to sports after ACL reconstruction, some of the assessment items are similar to the DIPCA.3, such as confidence in achieving good results in sports and being relaxed and ready to take on sports.^22^ This leads us to believe that the DIPCA.3 may be related to the IKDC subjective score. If the preoperative DIPCA.3 can be used predict knee joint function at 6 months after ACL reconstruction, this would be very useful information to facilitate return to sports. Athlete with high psychological competitive ability demonstrate high motivation, self-control, and concentration.^14^ These may be factors in achieving good knee joint function at 6 months postoperatively. Furthermore, these psychological states are likely to have a positive impact on returning to sports. However, there are no studies using DIPCA.3 assessment of psychological competitive ability in patients who underwent ACL reconstruction, so its usefulness is unknown.

Based on these considerations, two aims were identified for this study. First, to investigate the relationship between preoperative psychological competitive ability and preoperative and 6 months postoperative subjective knee function in patients undergoing ACL reconstruction. Second, to determine whether preoperative psychological competitive ability and subjective knee function are associated with return to participate in the entire practice at 6 months after ACL reconstruction. We hypothesized that preoperative psychological competitive ability would positively correlate with subjective knee function, and that those who were able to return to participate in the entire practice at 6 months after ACL reconstruction would have higher preoperative psychological competitive ability and preoperative and 6 months postoperative subjective knee function.

## 2 Methods

### 2.1 Participants

To maintain the quality of this study, ‘Strengthening the Reporting of Observational Studies in Epidemiology’ (STROBE) statement was used as a reference.

**Fig. 1:**
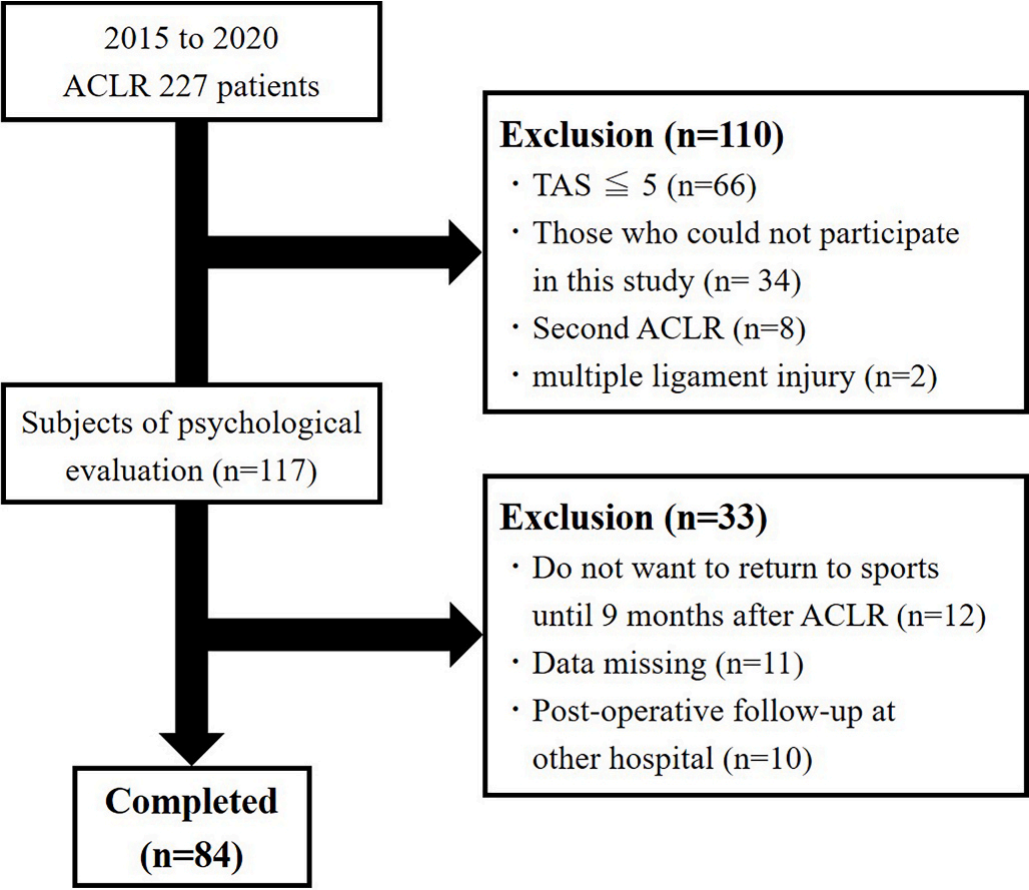
Flow chart of patient recruitment into this study.

We enrolled 227 patients who underwent ACL reconstruction at our hospital or at a hospital affiliated with ours between September 2015 and January 2020. All patients underwent ACL reconstruction using the hamstring tendon. The inclusion criteria were primary ACL reconstruction, Tegner activity level scale ≥6, and ability to undergo psychological evaluation before and 6 months after ACL reconstruction. The exclusion criteria were multiple ligament injury and re-reconstruction. All patients with missing data were also excluded. A total of 84 patients met these criteria and were included in this study (Figure 1).

This study was preliminarily conducted using G*Power 3.1.9.4 (Franz Paul, Kiel, Germany) to determine the power. To estimate the required sample size, an a priori power analysis was performed with the correlation: bivariate normal model. We found that 84 patients were needed for an effect size (*ρ*) of 0.3, an α level of 0.05, and a power of 0.8.^23^

### 2.2 Psychological assessment and return to practice

This prospective research was approved by the institutional review boards of the institution (1860). All patients gave informed consent for participation in this study, and consent signatures were obtained after hospitalisation for ACL reconstruction. The DIPCA.3 and the IKDC subjective score were used to assess the patient's psychological state. This study was a two-wave prospective study, and the evaluation periods were preoperative and 6 months after ACL reconstruction. Preoperative evaluations were performed after admission for surgical purposes with the assessments for the DIPCA.3 and IKDC subjective score. In addition, the assessment for IKDC subjective score was performed at the outpatient visit at 6 months postoperatively. These psychological evaluations were conducted in the rehabilitation room.

The DIPCA.3 was developed by Tokunaga et al.^8^^,^^9^^,^^14^ and comprises 5 factors, 12 subscales, and 52 question-based items (Table 1). Each of the 52 questions was answered on a 5-point scale from 1 to 5; 1) Not at all (0–10 %), 2) Seldom so (25 %), 3) Occasionally so (50 %), 4) Frequently so (70 %), and 5) Always so (90–100 %). The maximum score for each subscale is 20 points the total score ranges from 48 to 240 points, with higher scores indicating better psychological competitive ability (Table 1). Psychological competitive ability is graded from 1 to 5 from the DIPCA.3 total score; 1) poor (men: 141 or lower, female: 131 or lower), 2) fair (men: 142–164, female: 132–154), 3) good (men: 165–186, female: 155–178), 4) superior (men: 187–209, female: 179–202), 5) excellent (men: 210 or more, female: 203 or more).^8^^,^^9^^,^^14^

The IKDC subjective score evaluates the condition of the knee in terms of symptoms, the performance of sports activities, and function. The results are presented on a scale of 0 %–100 %, with higher scores indicating better knee function.^16^ At 6 months postoperatively, the patients were also surveyed on whether they could return to participate in the entire practice. The criteria for return to participate in the entire practice were based on the results of a previous study.^24^ Participation in the entire practice shown in this study excludes game-style practices and refers to other practice programs. Patients were classified into two groups according to whether they could return to participate in the entire practice (Successful return to practice and No return to practice), and then compared. The comparison items were the preoperative DIPCA.3 total score and IKDC subjective score preoperatively and 6 months after ACL reconstruction.

### 2.3 Rehabilitation and goals

Most patients received perioperative physical therapy according to the same rehabilitation protocol. Depending on the extent of the meniscus damage, their surgeons ordered two weeks of immobilisation in the extended position and non-weight bearing. The postoperative rehabilitation goals were as follows: (1) A knee ROM of 0°–90° at 2 weeks after ACL reconstruction. (2) A knee ROM of 0°–120° at 4 weeks after ACL reconstruction. Closed kinetic chain muscle strengthening exercises such as half squats and lunges were started. (3) A limb symmetry index (LSI) of ≥60 % at 3 months after ACL reconstruction, calculated based on the results of isokinetic knee extensor and flexor muscle strength measurement; if this was achieved, jogging was started. (4) An LSI of ≥90 % at 6 months after ACL reconstruction, at which point the patient returned to sport (return to official competition after game-style practices). The rehabilitation protocol did not include a specific psychological approach.

### 2.4 Statistical analysis

All analyses were performed using the Statistical Package for the Social Sciences software (version 27; IBM Corp, Chicago, IL, USA).

For the first aim, multiple regression analysis was performed with the preoperative IKDC subjective score as the dependent variable and DIPCA.3 factor scores as the independent variables. The five major items of DIPCA.3, such as “Volition for competition”, “Mental stability and concentration”, “Confidence”, “Strategic ability”, “Cooperation” were used as independent variables and analyzed using the forced entry method. DIPCA.3 grade scores were used in all DIPCA.3-related analyses.

For the second aim, the total DIPCA.3 score and IKDC subjective score were compared between participants with and without successful return to sport using the Student's *t*-test.

In addition, Cronbach's alpha was determined to demonstrate the reliability of the DIPCA.3 score used in this study.

## 3 Results

The basic characteristics of the participants are shown in Table 2. The total and grade scores for each DIPCA.3 factor are shown in Table 3.

The results regarding the first aim were as follows and rejected our hypothesis. The results of the multiple regression analysis showed that the preoperative DIPCA.3 Confidence score was related with the preoperative IKDC subjective score (β = −0.34, p = 0.04, r^2^ = 0.13; Table 4).

The results regarding the second aim were as follows and were partially consistent with the hypotheses. The return-to-sport rate 6 months after ACL reconstruction was 53.6 % (45/84 patients). There were no significant differences in the total DIPCA.3 score and preoperative IKDC subjective score between participants with and without successful return to practice (Table 5). However, the IKDC subjective score 6 months after ACL reconstruction was significantly higher in participants who successfully returned to the practice than in those who did not (Table 5).

The value of Cronbach's alpha for the DIPCA.3 score was 0.78 and was within the reference range of a previous study.^25^

## 4 Discussion

This study is the first to examine the relationship between psychological competitive ability and subjective knee function in patients undergoing ACL reconstruction. The results of this study completely rejected the first hypothesis and were partially consistent with the second. Notably, athletes with higher preoperative confidence had lower preoperative subjective knee function, but preoperative psychological competitive ability was not related to subjective knee function 6 months after ACL reconstruction. Furthermore, the preoperative psychological competitive ability was not associated with return to participate in the entire practice at 6 months postoperatively.

The DIPCA.3 Confidence score assesses an athlete's confidence in their abilities and in their ability to achieve their goal.^14^ The DIPCA.3 also includes judgement as a subscale, which includes items on the ability to play boldly and make decisions without fear of failure. Previous studies have shown that as the confidence of an athlete in their abilities increases, their level of active participation in the competition and their motivation to achieve a high level of performance also increases.^26^^,^^27^ These findings suggest that athletes with high confidence might have sought to achieve a high level of performance even before ACL reconstruction, and they had a low subjective knee function score because their knee function was insufficient to achieve the level of performance they sought to achieve.

This finding is important because it has been reported that the return-to-sport rate is high when both psychological state and subjective knee function are well controlled.^21^^,^^28^ However, no correlation was found between preoperative psychological competitive ability and subjective knee function at 6 months postoperatively. This suggested the possibility that an assessment tool focused on psychological status during sports activities, such as psychological competitive ability, may not be a good predictor of subjective knee function and return to sports 6 months after ACL reconstruction. On the other hand, a recent systematic review of preoperative predictors of return to sports activity after ACL reconstruction emphasizes the importance of psychovitality and appropriate goal setting.^29^ A similar item to psychovitality exists in the DIPCA.3 “Volition for competition” which includes the subscales of patience and volition for self-realization. A high level of these abilities indicates a superior ability to pursue one's goals in competition. Since the duration of rehabilitation after ACL reconstruction is long, it is important to set appropriate goals on a regular basis; thus, it may be necessary to conduct studies focusing on the ability to carry out goals.

There was a statistically significant improvement in the IKDC subjective score from before to 6 months after ACL reconstruction. An IKDC subjective score >85 points is often used as a criterion for satisfactory recovery.^28^^,^^30^ Here, the mean IKDC subjective score 6 months after ACL reconstruction was 81.0 ± 12.3 points, which is less than the aforementioned cut-off value. The postoperative IKDC subjective score has been reported to be related to return to participate in the entire practice in patients undergoing ACL reconstruction.^31^^,^^32^ Most previous studies that reported good subjective knee function assessed it approximately 12 months after ACL reconstruction.^5^^,^^21^ In contrast, we evaluated it at 6 months, which may have attributed to the discrepancy in the results. In a study by Agarwalla et al.^19^ 722 patients who underwent ACL reconstruction had a mean improvement in IKDC subjective knee function of 5.9 points at 6–12 months postoperatively. Therefore, we believe that our patients would have shown satisfactory recovery in IKDC subjective score if they had been evaluated 12 months after ACL reconstruction. Moreover, the IKDC subjective score of the participants who successfully returned to the practice 6 months after ACL reconstruction was 85.4 ± 9.8 points, which is similar to the recommended score.^28,30^As mentioned above, the course of subjective knee joint function of the subjects in this study was considered on average good. However, throughout the current study, no correlation was found between the preoperative DIPCA.3 results and the IKDC subjective score at 6 months postoperatively or with return to participate in the entire practice. Participation in the entire practice in this study is an earlier endpoint compared to return to sport outcomes. It is also a lower hurdle than the final goal of return to sport. The possibility that these timings and phasing levels led to the results of this study cannot be ruled out and will be investigated in the future.

This study has several limitations. First, this study uses self-report of evaluations, which is one of the weaknesses of the present research methodology. Second, the DIPCA.3 only evaluates preoperative psychological competitive ability, and its score does not reflect changes at 6 months after ACL reconstruction. Third, the patients did not receive any psychological intervention. It is unclear how the inclusion of a psychological approach to rehabilitation would have affected the results of this study, and future studies should include collaborations with sports psychologists and clinical psychologists. Fourth, the effect of limited early postoperative motion following meniscus repair on these results is unknown. Fifth, the analysis mainly uses correlation analysis, and no detailed statistical analysis is available. Finally, the postoperative follow-up period was short, and future studies should include long-term follow-up.

## 5 Conclusions

Preoperative psychological competitive ability negatively correlated with preoperative subjective knee function, but not with 6-month postoperative subjective knee function, in patients who underwent ACL reconstruction. Furthermore, the psychological competitive ability is not associated with a return to participate in the entire practice, and a low preoperative IKDC subjective score should not be viewed too negatively.

## Declaration of competing interest

None to declare.

## Figures and Tables

**Table 1:** DIPCA.3 factors and subscales

Factors	Subscales	Total score
Volition for competition	Patience	80 points
	Agressiveness	
	Volition for self-realization	
	Volition for winning	
Mental stability and concentration	Self-control	60 points
	Ability to relax	
	Concentration	
Confidence	Confidence	40 points
	Decision	
Strategic ability	Predictive ability	40 points
	Judgement	
Cooperation	Cooperation	20 points

Total score		240 points

DIPCA.3: Diagnostic Inventory of Psychological Competitive Ability for Athletes.

Each subscale has four questions and is rated on a five-point scale. 1) Not at all (0–10 %), 2) Seldom so (25 %), 3) Occasionalo (50 %), 4) Frequently so (70 %), and 5) Always so (90–100 %).

**Table 2:** Basic characteristics of the participants

Basic characteristics	
Sex (male:female)	(34:50)
Age (years)	21.1 ± 9.5
Height (cm)	165.7 ± 8.9
Weight (kg)	61.5 ± 12.4
BMI (kg/m^2^)	22.3 ± 3.2
TAS score	8.0 (6–9)
Preoperative IKDC subjective score	58.6 ± 14.4
Six-months postoperative IKDC subjective score	81.0 ± 12.3

BMI: Body mass index, TAS: Tegner Activity Scale, IKDC: International Knee Documentation Committee.

Age, height, weight, and BMI are presented as mean ± SD.

TAS score is presented as median (range).

**Table 3:** Mean DIPCA.3 factor scores

Factors	Total score	Grade score
Volition for competition	58.8 ± 11.1	3(1–5)
Mental stability and concentration	44.1 ± 9.5	3(1–5)
Confidence	23.6 ± 7.1	3(1–5)
Strategic ability	23.5 ± 7.0	3(1–5)
Cooperation	15.8 ± 3.3	3(1–5)

Total	166.5 ± 28.1	3(1–5)

DIPCA.3: Diagnostic Inventory of Psychological Competitive Ability for Athletes.

Total scores are presented as mean ± standard deviation. Judgement scores are presented as median (range).

The grade score ranges from 1 to 5. The higher the score, the better the psychological competitive ability.

**Table 4:** Relationship between psychological competitive ability and preoperative subjective knee function on multiple regression analysis

Model	Variable	Unstandardized	coefficients	Standardized coefficients	p-value	Adjusted R^2^
		B	SE	β		
Preoperative IKDC subjective score	Volition for competition	1.00	2.00	0.07	0.62	0.07
	Mental stability and concentration	2.60	1.48	0.21	0.08	
	Confidence	−4.31	2.12	−0.34	0.04	
	Strategic ability	−1.55	2.16	−0.12	0.48	
	Cooperation	0.42	1.41	0.04	0.77	

IKDC: International Knee Documentation Committee, SE: Standard error.

Degree of freedom = 4, Statistical significance was set at p = 0.05.

**Table 5:** Comparison of psychological competitive ability and subjective knee function with return to practice at 6 months after anterior cruciate ligament reconstruction

	Successful return to practice	No return to practice	p-value
DIPCA.3 total score	166.0 ± 27.7	167.2 ± 28.5	0.84
Preoperative IKDC subjective score	57.8 ± 14.6	59.5 ± 14.2	0.60
Six-months postoperative IKDC subjective score	85.4 ± 9.8	75.9 ± 12.9	<0.01

DIPCA.3: Diagnostic Inventory of Psychological Competitive Ability for Athletes, IKDC: International Knee Documentation Committee.

Statistical significance was set at p = 0.05.
